# Cascading effects of polyphenol-rich purple corn pericarp extract on pupal, adult, and offspring of tobacco hornworm (*Manduca sexta* L.)

**DOI:** 10.1080/19420889.2020.1735223

**Published:** 2020-04-01

**Authors:** Mandeep Tayal, Pavel Somavat, Isabella Rodriguez, Leilani Martinez, Rupesh Kariyat

**Affiliations:** a Department of Biology, The University of Texas Rio Grande Valley, Edinburg, TX, USA; b School of Earth, Environmental, and Marine Sciences, The University of Texas Rio Grande Valley, Edinburg, TX, USA; c Mathematics and Science Academy, The University of Texas Rio Grande Valley, Edinburg, TX, USA; d Weslaco High School, Weslaco, TX, USA

**Keywords:** Tobacco hornworm, polyphenols, purple corn, herbivore, growth and development, pupal mass

## Abstract

A major bottleneck in the commercialization of plant-based pest management compounds is that the extraction methods are complex, time-consuming, and even highly expensive. Using a recently developed inexpensive extraction and quantification methodology to isolate polyphenols (including anthocyanins and condensed tannins) from purple corn pericarp, we examined their effects on *Manduca sexta*, a common insect herbivore. Following up on our previous work which demonstrated the negative impacts of polyphenol-rich extract on larval stages, we further examined whether there are any cascading effects on subsequent life stages (pupal and adult) including any possible transgenerational effects. Our results show that polyphenol-rich purple corn extract-fed caterpillars had significantly lower pupal mass and survival. Moreover, adult moths also had lower mass when eclosed from caterpillars reared on the extract diet. To test whether there were any transgenerational effects, we allowed male and female adults fed on purple corn extract diet and control diet to mate and lay eggs in a full factorial experiment. We found that purple corn extract-fed adult pair laid a lower number of eggs compared to other treatments. In addition, we also found that second instar *M. sexta* caterpillars hatched from eggs laid by any mating combination with at least one parent reared on purple corn extract gained significantly lower mass compared to caterpillars with both parents reared on the control diet. Taken together, our results show that there are cascading negative effects for feeding purple corn pericarp extract on pupal, adult, and second generation of *M. sexta*, reaffirming its potential application as a cost-effective and environmentally friendly pest deterrent.

## Introduction

Globally, insect pests account for ~18% loss of total crop yield [1]. The use of synthetic insecticides/pesticides to manage these pests while delivering positive results has also produced a different set of concerns including resistance development, residue build up, biomagnification, and toxicity to non-target organisms [1–3]. Alternatively, a growing body of work has examined the insecticidal properties of plant-based bioactive compounds against various insect pests [4]. For example, Azadirachtin (C-*seco*- limotriterpenoid) extracted from neem plant *(Azadirachta indica)* has been shown to exhibit larvicidal effect against horn fly (*Haemotobia irritans*, L), stable fly (*Stomoxys calcitrans*) and house fly [*Musca domestica*; 5] as well as fifth instar larvae of tobacco hornworm [*Manduca sexta*; 6,7]. Generally, these compounds act as antifeedant, repellent, anti-ovipositor and in some cases as toxins that can impede feeding, negatively affecting growth and development or even kill insects [6,8].

To test their efficacy against insect pests, these compounds are generally used either as crude extracts or pure compounds [2,4,6,9,10]. However, due to the presence of diverse compounds in plant matrices, it becomes tedious to extract and purify these specific compounds efficiently and economically [11,12]. In other words, there is a never-ending quest for sourcing biologically active compounds that can satisfy the abovementioned properties to be potentially incorporated into sustainable pest management practices [4].

Colored corns (purple, blue) – are widely cultivated and consumed across Argentina, Bolivia, Ecuador, and Peru [13,14]. Purple corn (*Zea mays* L.) is considered to be one of the richest sources of polyphenols including anthocyanins and tannins [15]. Known for its antioxidant [16,17], anti-obesity [18,19], anti-cancer [16], as well as anti-inflammatory properties [20], these compounds (especially the combination of anthocyanins, polyphenols, and tannins) can potentially have multiple pharmacological uses. Since a lot of these plant metabolites are synthesized to boost plants’ defense mechanism, these also have potential insect deterrent/insecticidal properties [21–24]. For example, we recently reported that sorghum 3-Deoxyanthocyanidin flavonoids negatively affect feeding preference and reproduction of corn leaf aphid (CLA: *Rhopalosiphum maidis*) and provide resistance against CLA [21]. Although the precise mechanism behind these effects is less understood, these compounds, in general, have been shown to possess some insecticidal properties in various study systems [25–28].

Having longer storage life and the presence of significant amounts of such compounds [15], these pigmented corn varieties can potentially be explored as a source of inexpensive polyphenolic compounds for utilization as bioinsecticides. In addition, Somavat et al. [29] developed an efficient and inexpensive patent-pending [30] methodology to isolate them from corn pericarp, which is essentially a waste product in corn processing. This is indeed a significant step since, most of the current compound recovery methods from natural sources are plagued by complicated and expensive extraction methods, lower recovery of active ingredients, and also tend to be time-consuming [11,12,31].

In a previous study, we documented the negative effects of purple corn pericarp extract on growth and development of different larval stages of *Manduca sexta* [tobacco hornworm; Sphingidae; Lepidoptera; egg hatching, caterpillar mass, caterpillar mass gain, and time to pupation; 32]. We found that the purple corn pericarp extract added diet significantly decreased egg hatching percentage and lowered the mass and mass gain compared to control diets. Moreover, the purple corn pericarp extract-fed larvae showed significantly lower preference to feed on that diet compared to control diets, and took longer to pupate. We used *M. sexta* as a study system because of its size, rapid growth, ease of laboratory rearing, and their longtime use as a study system for physiological, developmental, and behavioral studies [33–38].

Although the larval stage of herbivorous insects such as *M. sexta* is very critical from crop husbandry perspective (caterpillars cause damage and adults are usually pollinators), it is imperative that other growth stages are also investigated to examine the possible lingering or cascading effects of feeding bioactive compounds to them. This is especially important since adult females can lay over 200 eggs in *M. sexta* [33], and oviposition is considered as the first sign of herbivory [39]. Moreover, it has been shown that the diet and energy requirements for holometabolous insects change between life stages [40]. In addition, the life cycle modularity and rapid compensation also allow insects to uncouple the effects of environmental disturbances on their physiology in one step to the next [41–44]. For example, the food impoverished individuals of Glanville fritillary butterfly (*Melitaea cinxia*) maintained their high fecundity rate through compensatory increased developmental time [45]. It has also been reported that insect maternal effects on second-generation offspring in response to parent diet quality are critical for their growth [40,46–50]. For example, in *Drosophila melanogaster*, the parents reared on poor larval food laid heavier but smaller eggs than control parents showing adaptive and maladaptive effects of parental stress [51]. In other words, it is plausible to expect that adults emerging from stressed caterpillars (in this case, the purple corn extract-fed specimen) when allowed to mate can possibly produce offspring that are compromised in their growth and development.

**Figure 1.**
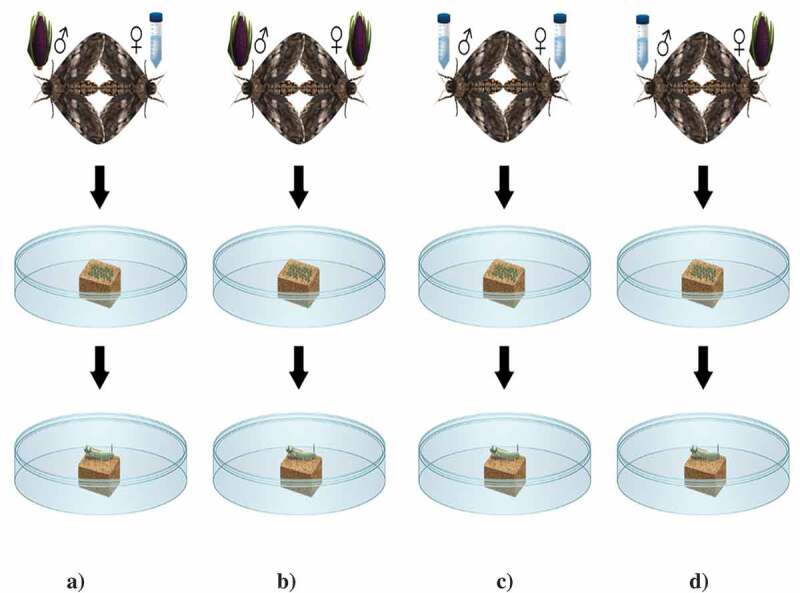
Schematic representation of the experimental design to study the effects of pericarp extract on fecundity and mass of first and second instar offspring larvae. Adult moths were crossed in all possible combinations (a) Control female × Purple male, (b) Purple female × Purple male, (c) Control female × Control male, and d) Purple female × Control male. All the collected eggs were counted and placed on an artificial control diet and were allowed to hatch. Caterpillar mass at first and second instar was measured and used to compute mass gain.

Keeping this in mind, we designed a set of experiments where we continued to examine the effects of feeding purple corn pericarp extract on different pupal (pupal mass, pupal survival, pupal duration) and adult (adult mass, adult wingspan) parameters. We also had two control diets; a regular artificial diet with no extract added, and an additional control diet with yellow corn extract added. To study whether there are any transgenerational effects of feeding polyphenol-rich pericarp extract on *M. sexta*, we allowed the controlled mating of adult moths on purple corn extract diet and control diet (artificial diet without extract) treatments in a full factorial design (see Figure 1 for details), allowed them to lay eggs, and their offsprings to grow.

## Material and methods

### Insect colony

The pupae and adult moths used in this study were collected from our previous experiment in which their respective larvae were allowed to feed on different treatments (purple corn pericarp extract: N = 58, yellow corn pericarp extract: N = 28, control: N = 51) at room temperature. At fifth instar stage, when the caterpillars stopped eating and started to wander in petri dishes with the dark black pulsating vein on dorsal side clearly visible (Supplementary video S1), they were transferred to plastic containers (23.19 cm × 15.24 cm × 16.84 cm; Aquaculture pet carrier: # 564356887, Walmart) with wood shavings (Natural Aspen small animal bedding: Petco Animal Supplies, Inc., San Diego, CA, USA) for pupation. Once pupated, measurements were taken, and the pupal containers were then moved to lab cabinets and kept under dark conditions at room temperature of 27°C and RH of 65%.

### Pericarp extract and its quantification

Pericarp extracts used in different caterpillar diet treatments (purple corn, yellow corn) were obtained by steeping 5 gm of respective pericarps in 100 ml of deionized water followed by stirring and centrifugation at 5000 rpm for 5 minutes [29]. The resultant filtrate was used for mixing in caterpillar food. A pH differential method using 96-well microplate reader (Multiskan Sky Microplate Spectrophotometer: #51119600, Thermo Fisher Scientific, MA, USA) was used to quantify the amount of total monomeric anthocyanins, polyphenols, and total tannins present in extracts. It was found that purple corn pericarp extract contained greater amounts of total monomeric anthocyanins, total polyphenols, and tannins compared to yellow corn pericarp extract which contained no anthocyanins and lower amounts of total polyphenols and tannins [52].

### Experiment methodology

All healthy pupae from the three different treatments (purple corn pericarp extract, yellow corn pericarp extract, and control) were allowed to develop and eclose. Mass of each pupae was recorded by weighing them on a digital balance (Accuris Series Dx, Model: W3100-210, Benchmark Scientific, NJ, USA). Once they eclosed, we calculated the days from pupation to eclosion, recorded as pupal duration (all data were collected at the same time to be consistent). Pupae that did not eclose after 45 days and stopped moving were considered as dead and were used to calculate the pupal survival. Once eclosed, we recorded the adult mass as well as wingspan. While adult mass was calculated by using a digital balance (Accuris Series Dx, Model: W3100-210, Benchmark Scientific, NJ, USA), adult wingspan, i.e., length from the tip of one wing to the tip of other wing when fully expanded, was measured with a ruler (Ruler, White Vinyl: # 70260, North Carolina Biological Supply Co., NC, USA).

To examine the transgenerational effects of pericarp extract, we mated adult moths from purple corn pericarp extract and control treatments to each other in all possible combinations (Purple female × Purple male; Purple female × Control male; Control female × Purple male; Control female × Control male; Figure 1 (N = 5-7/treatment), for mating details see [33]. Following the mating design, adult moths (one newly eclosed male and female each) were placed in popup cages (Popup rearing cage: #1466AB, BioQuip Products, Inc., CA, USA) along with orange-flavored Gatorade [33] as their food and a 6 weeks old tomato plant (Variety: Valley Girl, Product ID 741, Johnny’s Selected Seeds, ME, USA) as a host for oviposition [33]. Cages of different crosses were monitored every day for eggs until the females died. Collected eggs were moved to regular artificial control diet at room temperature [35]. Once the larvae hatched, we recorded larval mass to observe any transgenerational effects.

### Statistical analysis

We used Analysis of variance as well as the non-parametric Kruskal–Wallis tests depending upon the nature of data (normal or non-normal) for analysis. Since pupal duration followed normal distribution, we ran one-way Anova (diets as a factor) for analysis. However, non-normal data of pupal survival, adult wingspan, were analyzed with non-parametric Kruskal–Wallis test. Post-hoc pairwise comparisons of all treatments were obtained with Tukey’s HSD and Dunn’s multiple comparison tests, respectively. We also collected data on larval mass at two-time intervals – corresponding to first and second instars. Larval mass data were analyzed using Generalized regression with maximum likelihood estimation and Dunnett’s post-hoc tests were used to compare all pairwise combinations with control. For pupal and adult mass, we ran two-way Anova where both treatment (diets) and sex (male and female) were considered as factors and their interaction term was also added into the model. Data sets were analyzed using the statistical software, JMP (SAS institute, NC, USA), and plots were built using GraphPad PRISM software (La Jolla, CA, USA). Detailed statistics are described in Table 1.

**Table 1.** Statistical test details used to analyze the effect of different pericarp extracts on pupal, adult, and transgenerational traits. Significant results with p < 0.05 are in bold.

Parameter	Test	Df/groups	Test Statistics	p-value
Pupal mass	Two Way ANOVA	2	Treatment (diet) F = 23.88	**<0.0001**
		1	Sex (male and female) F = 7.65	**0.0067**
		2	Interaction F = 4.53	**0.013**
Adult mass	Two Way ANOVA	2	Treatment (diet) F = 14.58	**<0.0001**
		1	Sex (male and female) F = 6.97	**0.010**
		2	Interaction F = 1.73	0.183
Pupal survival	Kruskal-Wallis Test	3	Kruskal-Wallis Statistic = 9.88	**0.0071**
Pupal duration	One Way ANOVA	2	F statistic = 5.665	**0.0047**
Adult wingspan	Kruskal-Wallis Test	3	Kruskal-Wallis Statistic = 2.38	0.3037
Eggs laid	Kruskal-Wallis Test	4	Kruskal-Wallis Statistic = 4.83	0.1847
First instar larval mass	Generalized Regression	3	Wald Chi-Square = 9.56	**0.0226**
Second instar larval mass	Generalized Regression	3	Wald Chi-Square = 35.32	**<0.0001**

## Results

### *Effects of pericarp extract on pupal stage of* M. sexta

**Figure 2.**
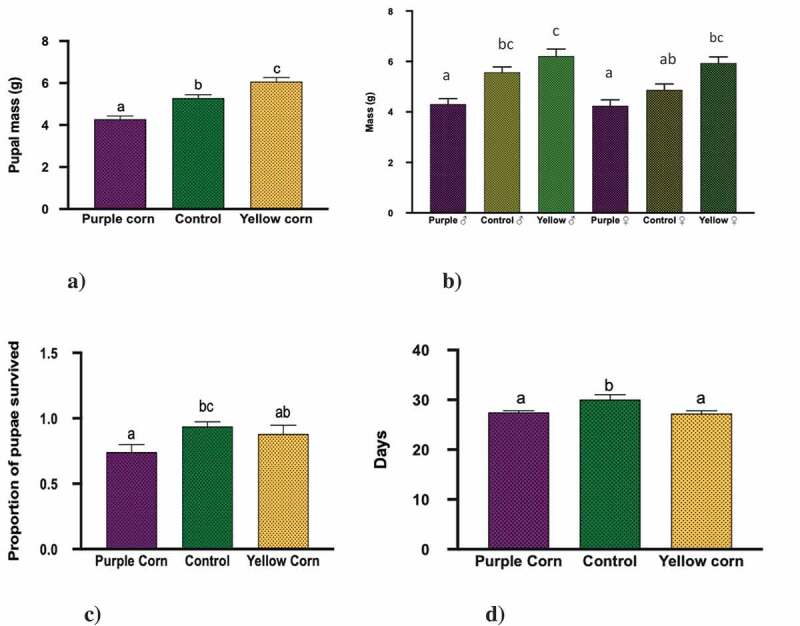
Results of one-way ANOVA, two-way ANOVA, Kruskal–Wallis tests, and the post-hoc Tukey’s HSD and Dunn’s multiple comparisons (p < 0.05) for the effects of pericarp extract diet on mean (a) pupal mass, (b) pupal mass: treatment × sex, (c) pupal survival, and (d) pupal duration. Means followed by different letters are significantly different at p < 0.05.

Both pupal and adult mass results showed similar trends. Results from the Two-Way Anova for pupal mass showed that all the main effects were significant; diet treatment (F = 23.88, p < 0.0001), sex (F = 7.65, p = 0.0067), and interaction (sex × treatment; F = 4.53, p = 0.01) (Table 1). Pairwise comparisons showed that pupae from caterpillars reared on yellow corn diet were significantly heavier than both control and purple corn (Figure 2(a)). We also found that regardless of the treatments, male pupae from yellow corn and control diets were heavier than purple corn and control female pupae, which was surprising (Figure 2(b)).

Pupal survival was significantly low (F = 9.885, p = 0.0105) for purple corn reared pupae compared to control (Figure 2(c)). However, yellow corn reared pupal survival was similar to purple corn and control pupae (F = 9.885, p = 0.0852). Interestingly, we found that pupae reared on purple and yellow corn diet had significantly low pupal duration (in days) (F = 5.665, p = 0.0047) and eclosed earlier compared to control pupae (Figure 2(d)).

### *Effects of pericarp extract on adult stage of* M. sexta

**Figure 3.**
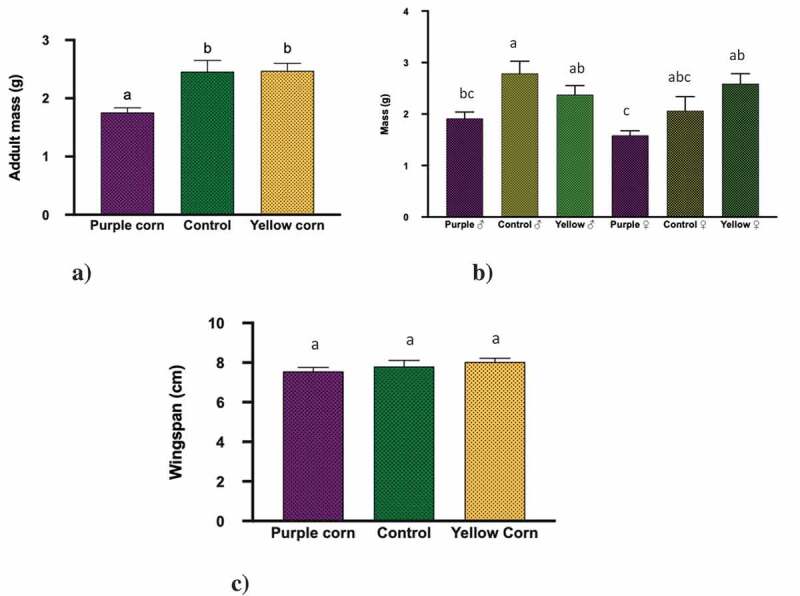
Results of one-way ANOVA, two-way ANOVA, Kruskal–Wallis tests, Post-hoc Tukey’s HSD test (p < 0.05) for the effect of pericarp extract diet on mean (a) adult mass, (b) adult mass: treatment × sex, and (c) adult wingspan. Means followed by different letters are significantly different at p < 0.05.

For adult mass, we found that both treatments (diets; F = 14.15, p < 0.0001 and sex; F = 6.971, p = 0.010) significantly affected the mass, while their interaction was non-significant (F = 1.73, p = 0.183) (Table 1). Upon close examination with pairwise comparisons, we found that adults emerged from caterpillars fed on yellow corn diet and control diet were significantly heavier than the ones from purple corn diet (Figure 3(a)). In addition, we also found that regardless of the treatments, male moths were heavier compared to females (Figure 3(b)). However, there was no significant difference in wingspan (F value = 2.38, p < 0.3037) across the treatments (Figure 3(c)).

### Effect of pericarp extract on adult fecundity and offspring mass

**Figure 4.**
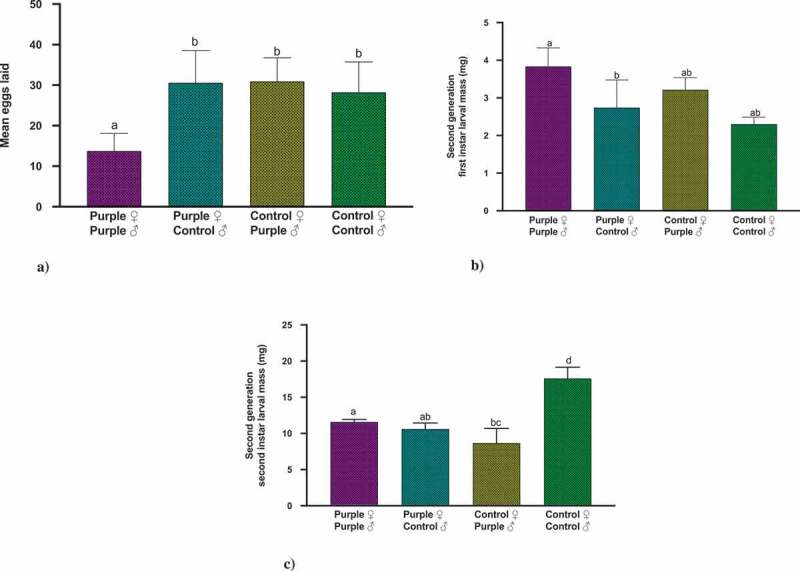
Results of the Kruskal–Wallis tests, Dunn’s multiple comparison test (p < 0.05) for the effect of pericarp extract diet on (a) mean number of eggs, (b) first instar offspring larval mass, and (c) second instar offspring larval mass. Means followed by different letters are significantly different at p < 0.05.

Although there were no significant differences (F value = 4.83, p = 0.1847) in mean number of eggs laid by different crosses, a pattern of lower number of eggs laid was observed when both the parents were fed on purple corn pericarp extract diet (Figure 4(a)). More interestingly, when we examined the larval mass in the second generation, we found that for first instar, the treatment was statistically significant (Wald Chi-Square = 9.56, p = 0.0226), but when we used Dunnett’s post-hoc comparison tests to compare the male-female combinations with Control female × Control male, none of the combinations were statistically significant (Figure 4(b)). However, once the caterpillars became second instar, these differences were amplified. Again, the treatment was significant (Wald Chi-Square = 35.32, p < 0.0001). More interestingly, Dunnett’s pairwise comparisons revealed that all pairwise combinations, when compared to Control female × Control male were statistically significant, clearly demonstrating that the presence of any of the parents reared on purple corn pericarp extract diet affected the offspring (Purple female Control male with Control male Control female; p = 0.002; Purple male Purple female with Control male Control female; p = 0.025; and Control female Purple male Control male Control female; p <.0001). (Figure 4(c))

## Discussion

In continuation of our recent study demonstrating the negative effects of purple corn pericarp extract on different larval stages (egg hatching, larval mass, mass gain and feeding preference, etc.) of *M. sexta* [52], current study clearly demonstrates similar effects cascading through pupal, adult, and next-generation offsprings. The lower mass observed in purple corn pericarp pupae is a direct result of purple corn pericarp-fed larvae that had gained lower larval mass, clearly indicating larval nutritional stress had negative effects on subsequent life stages. Similar results have been reported on lower pupal mass of starved caterpillars, for example, in Squinting bush brown (*Bicyclus anynana*), which signifies the importance of diet on their post-larval life history traits [32,40,53–56].

The significant decline in pupal survival and developmental time of purple corn pupae directly affected adult mass and fecundity similar to predictioned by life-history models [40,57–60]. It is well documented that juvenile and ecdysteroid hormone levels direct postembryonic insect development [61], we speculate that purple corn pericarp extract might have affected the interendocrine regulation, resulting in possible low survival rates. In our recent work, we found a large number of larvae stayed away from pericarp extract diet [52] and were reluctant to feed on it compared to control diets. It is possible that polyphenol-rich diet may have antifeedant properties which resulted in starvation (or reduced intake) and consequently decreased larval and pupal mass.

In addition, the findings of decreased pupal duration are consistent with previous studies which have also documented reduced lifespan in Glanville fritillary butterfly (*Melitaea cinxia*) due to early larval food stress [45]. On the other hand, reduced lifespan and faster development may also result in poor growth which can affect subsequent stages. Parallel to this assumption, we found significantly lower mass for adults of purple pericarp extract group compared to control diet and yellow corn pericarp extract group, reflecting their inability to compensate. These findings are also in agreement with [62] and many others, who reported a strong correlation between pupal mass and adult mass at eclosion. The presence of reduced mass pattern in larval, pupal, and adult mass show cascading negative effects of early larval food stress on subsequent stages [32,40,52].

While examining the sex-specific effects of pericarp extract on male and female individual masses, lack of any significant differences among treatment × sex interaction indicates that these effects are sex-independent. In 2005, Bauerfeind & Fischer also did not find any significant interactions among larval food stress treatments and sex in Squinting bush brown (*Bicyclus anynana*) although, the female pupae had higher mass than male pupae. Previous studies have reported that the adverse effects of poor diet quality are more severe if both the parents and earlier generations experience them, as demonstrated in this study [50,63,64]. Having a strong correlation of pupal mass on female longevity, fecundity, and egg size [56], it is understandable that low female pupal mass would directly affect their reproductive ability.

Moreover, in relation to the individual size and mass, we also expected that polyphenol-rich pericarp extract-fed adults may have smaller wings and hence lower wingspan [65] although, the results were not on expected lines. However, since wings are an important part for foraging, dispersal, and mate searching [66], it would be interesting to study these effects on molecular level, i.e., muscle molecular composition and their flight capacity [67,68]. We speculate that there could be possible effects of polyphenol-rich pericarp extract on the alternate splice forms of Troponin T [67].’

Although we did not find any significant differences in mean number of eggs laid among different crosses, when both parents belonged to purple corn pericarp extract diet, they indeed had the lowest number of eggs. Number of eggs (123 out of total 905, ~13%) demonstrated the effects of early larval stress on fecundity – possibly due to poor food quality that affected insect physiology and fitness for reproductive investment, coupled with short pupal development [40,48–50]. Reduced sample size per treatment (~24 mating pairs) and high variability in egg laying (ranging 0 to 447) might explain the reasons we could not resolve many possible pairwise differences for egg laying in this study. Since oviposition is the first step in future herbivory and herbivory-related defense induction [69,70–72], a reduction in fecundity of adults could be a management strategy against herbivory [73,74].

Interestingly, the low second instar larval mass of offsprings from a mating where at least one parent was from pericarp extract diet confirms the transgenerational effects of purple corn pericarp extract. In the past, different studies have shown the role of quality nutrition in female reproduction and indirectly, its negative transgenerational effects [40,75–79]. In contrast, the fitness buffering ability of individuals may also result in adaptive responses against sensitive environmental stresses [50,65,80–82]. For example, it has been reported that mothers exposed to stress through natural enemies may produce more resistant offsprings [45,80]. However, our results are in contrast to this response and demonstrated decreased offspring mass of nutritionally stressed parents. Also, as neonates have to cope with different factors such as plant surfaces, plant defenses, predators, pathogens, and parasitoids to establishing themselves on food plant [34,83,84], we speculate that the reduction in early instar larval masses makes them more susceptible. Future studies should be focused to identify whether any adaptive effects (any resistant individuals) of pericarp nutritional stress are transmitted to next generations.

Plants produce a variety of secondary metabolites (alkaloids, terpenes, phenolics, nitrogen, and sulfur-containing compounds) for defending themselves against herbivores either by direct toxicity or by indirectly attracting their parasites and predators [36,85–90]. It has also been shown that these defenses are upregulated in response to chemical elicitors present in herbivore and pathogen oral secretions [91]. For example, Cai et al. [92] reported an increased level of anthocyanins and resveratrol in *Vitis vinifera* cell suspension cultures in response to *M. sexta* saliva, suggesting the possibility that anthocyanins are also a part of herbivore defense mechanism, and are inducible, suggesting an evolutionary history of such compounds in plant defense, further supporting the merits in our exploration of these extracts for pest management.

## Conclusions and future studies

It was found that polyphenol-rich purple corn extract, an inexpensive byproduct of corn processing has negative impact on pupal and adult fitness and these effects can cascade through transgenerational stages of *M. sexta*. In previous work, we found the negative effects of this extract on egg hatching, caterpillar mass, developmental time [52], suggesting anti-herbivore effects of polyphenolic compounds and its overall suitability as a biopesticide. Associated complexities and difficulties in bioactive compound extraction mainly limit their commercialization potential. However, polyphenol-rich purple corn extract can prove to be an economically viable insect deterrent/biopesticide. In addition, since purple corn pericarp extract is a diverse mix of anthocyanins, tannins, and other polyphenols, additional research is needed to ascertain whether these effects are due to a specific bioactive compound or due to a synergistic effect of all these compounds. In addition, apart from lepidoptera pests, it would be interesting to test the efficacy of polyphenol-rich purple corn extract against pests with different feeding habits, i.e., aphids, white flies, etc. Finally, future studies are required to identify the mechanisms/mode of action of polyphenol-rich pericarp extract at molecular levels, an area we are currently exploring.

## Data Availability

Data is available online in the Dropbox (https://www.dropbox.com/s/lorty5vyvt5h9sj/CIB%20Data%202020.xlsx?dl=0).
